# A Case of Peritoneal Dialysis-Related Peritonitis Due to Moraxella osloensis

**DOI:** 10.7759/cureus.74294

**Published:** 2024-11-23

**Authors:** Kenta Torigoe, Ai Yoshidome, Emiko Otsuka, Kiyokazu Tsuji, Ayuko Yamashita, Mineaki Kitamura, Takahiro Takazono, Noriho Sakamoto, Kumiko Muta, Hiroshi Mukae, Tomoya Nishino

**Affiliations:** 1 Department of Nephrology, Nagasaki University Hospital, Nagasaki, JPN; 2 Respiratory Medicine, Nagasaki University Graduate School of Biomedical Sciences, Nagasaki, JPN

**Keywords:** antibiotic therapy, end stage renal disease (esrd), moraxella osloensis, peritoneal dialysis, peritonitis

## Abstract

A 46-year-old woman on peritoneal dialysis (PD) had cloudy peritoneal dialysis effluent that had persisted for 10 days by the time she visited our hospital. The white blood cell count in the effluent was elevated to 1500/μL, leading to a diagnosis of peritoneal dialysis-associated peritonitis. The effluent cleared within two days with treatment using cefazolin and ceftazidime, and the white blood cell count dropped to 0/μL by day 6. Culture of the effluent revealed the presence of *Moraxella osloensis*. The patient’s treatment was switched to ceftazidime monotherapy, and antibiotic therapy for 21 days resulted in the resolution of the peritonitis. Reports of peritonitis caused by *Moraxella osloensis* are rare; however, with the spread of diagnostic methods, such as matrix-assisted laser desorption ionization-time of flight mass spectrometry, an increase in reported cases is expected. Unlike previous cases, in this case, the interval from onset to treatment initiation was longer. However, similar to reported cases, in this case, the infection was cured with antibiotic treatment without the need for PD catheter removal.

## Introduction

Peritonitis is a serious complication of peritoneal dialysis (PD) and is associated with the potential need to discontinue PD and increased mortality [1]. In Japan, the most common causative pathogens of PD-related peritonitis are Gram-positive cocci, accounting for 39% of all cases though cases of PD-associated peritonitis caused by other bacterial species have been reported [2]. Optimal treatment of this complication requires the selection of the most effective antibiotics based on the causative organism. Furthermore, prompt removal of the PD catheter is essential in cases in which specific pathogens are resistant to treatment. Therefore, accumulating clinical data on PD-related peritonitis caused by various pathogens is needed.

*Moraxella osloensis* (*M. osloensis*) is an aerobic, gram-negative bacillus commonly found on human skin and mucosal surfaces and is considered part of the normal respiratory flora [3,4]. Although generally low in pathogenicity, it has been reported to cause infections, such as sepsis, meningitis, and endocarditis, in immunocompromised individuals [5-7], and there have been a few reports of PD-related peritonitis. Herein, we report a case of PD-related peritonitis caused by *M. osloensis*. Unlike in previous cases, in this case, treatment initiation was delayed; however, favorable outcomes were achieved with antibiotic therapy.

## Case presentation

**Figure 1:**
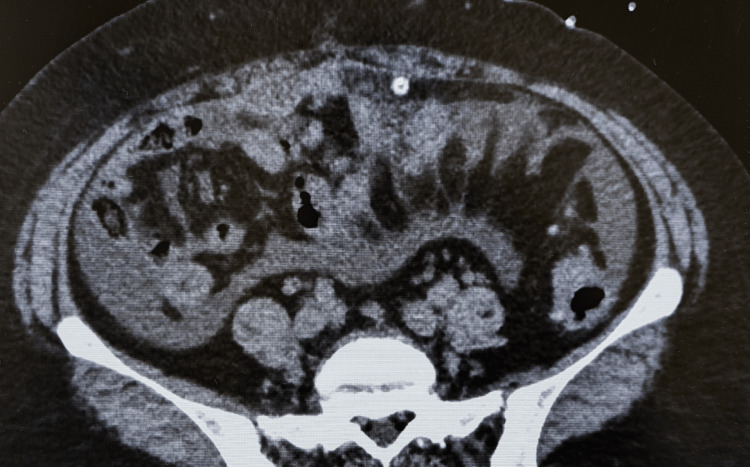
Computed tomography findings There are no notable abnormalities except for the presence of the PD catheter. PD: peritoneal dialysis

The patient was a 46-year-old woman who had been undergoing PD for end-stage renal disease due to chronic glomerulonephritis for seven years. She noticed cloudy PD effluent 10 days before presenting to our hospital; however, because she did not experience any abdominal pain or develop a fever, she did not contact her physician. On the day of her scheduled outpatient visit to our hospital, the cloudy PD effluent persisted. Her vital signs were as follows: body temperature, 36.8 °C; blood pressure, 170/100 mmHg; pulse rate, 66/min; and oxygen saturation (SpO₂), 99% (room air). Physical examination revealed no abdominal tenderness and no signs of infection at the PD catheter exit site or tunnel. Blood test results were as follows: white blood cell (WBC) count, 7500/μL; neutrophils, 82.7%; blood urea nitrogen level, 48 mg/dL; and creatinine level, 9.67 mg/dL. The WBC count in the PD effluent was elevated to 1500/μL (neutrophils, 90%), leading to a diagnosis of PD-related peritonitis. Abdominal computed tomography performed later showed no notable abnormalities except for the presence of the PD catheter (Figure 1).

Because immediate hospitalization was not feasible, outpatient treatment with intravenous cefazolin (1 g/day) and ceftazidime (1 g/day) was initiated. Two days later, the patient was admitted to our department. Upon admission, cefazolin and ceftazidime were administered intraperitoneally at a dose of 1 g/day. On the day of admission, the cloudiness of the PD effluent had resolved, and the WBC count in the fluid had improved to 200/μL. On the fourth day of hospitalization, M. osloensis was identified in the PD effluent culture (by using matrix-assisted laser desorption ionization-time of flight mass spectrometry (MALDI-TOF MS)). Based on this result, the antibiotic regimen was adjusted to intraperitoneal ceftazidime 1 g/day (Table 1). Because the WBC count in the PD effluent decreased to 0/μL on the fourth day of hospitalization, the patient was discharged on the fifth day with the treatment to be continued on an outpatient basis. Ceftazidime was administered for 21 days, with no recurrence of PD-related peritonitis thereafter.

**Table 1:** Drug susceptibility test results for Moraxella osloensis MIC: minimum inhibitory concentration

Antimicrobial agent	MIC (mg/mL)
Penicillin G	8
Ampicillin	4
Sulbactam/ampicillin	≤0.5
Cefotaxime	≤0.5
Ceftazidime	≤0.5
Ceftriaxone	≤0.5
Cefepime	≤0.5
Imipenem	≤0.12
Meropenem	≤0.06
Erythromycin	0.25
Clarithromycin	≤0.5
Clindamycin	2
Vancomycin	>4
Levofloxacin	≤0.5
Linezolid	>4
Daptomycin	>1

## Discussion

We describe a case of a patient with PD-related peritonitis caused by *M. osloensis*. Unlike previously reported cases, in this case, the period from peritonitis onset to treatment initiation was prolonged. However, the peritonitis was cured with antibiotic therapy alone, without requiring PD catheter removal.

*M. osloensis* is an aerobic, gram-negative bacterium that colonizes human skin and mucous; it is also considered part of the normal respiratory flora [3,4]. Identification of *M. osloensis* has traditionally been challenging with conventional methods; however, the recent use of advanced identification technologies, such as MALDI-TOF MS, has led to an increase in reported cases [7]. To date, there have only been four reported cases of PD-related peritonitis caused by M. osloensis. However, further reports are anticipated, and clinical data on the characteristics of PD-related peritonitis due to M. osloensis must be accumulated.

Table 2 shows the details of cases of PD-related peritonitis caused by *M. osloensis*, including this case [8-11]. Resolution was achieved in all cases, although in one case, which was complicated by *Rhizobium radiobacter* infection, PD catheter removal was required. However, in cases of *M. osloensis* infection alone, including this one, peritonitis resolved with antibiotic therapy alone, without the need for catheter removal. Notably, the interval from onset to treatment initiation was longer in this case than in other cases. However, the patient still responded rapidly to treatment, reinforcing that PD-related peritonitis caused by *M. osloensis* generally has a favorable prognosis.

**Table 2:** Cases of PD-related peritonitis due to Moraxella osloensis ESRD: end-stage renal disease; PD: peritoneal dialysis

Reference	Age/sex	ESRD cause	Other bacteria cultured	Clinical findings	Period from onset to treatment (days)	Antibiotic treatment	Catheter removal	Outcome
8	83/Male	Unknown	None	Abdominal pain, cloudy peritoneal fluid	2	Cefazolin ＋ ceftazidime ＋ linezolid to ceftazidime ＋ amoxicillin	No	Cured
9	47/Male	Membranous nephropathy	Rhizobium radiobacter	Abdominal pain, fever, cloudy peritoneal fluid	Unknown	Cefazolin ＋ ceftazidime ＋ ciproﬂoxacin to meropenem	Yes	Cured
10	68/Male	Diabetic nephropathy	None	Abdominal pain, cloudy peritoneal fluid	1	Cefazolin	No	Cured
11	26/Male	Malignant nephrosclerosis	None	Abdominal pain, fever, nausea, vomiting, cloudy peritoneal fluid	2	Cefazolin ＋ ceftazidime to cefazolin	No	Cured
This case	46/Female	Chronic glomerulonephritis	None	Cloudy peritoneal fluid	10	Cefazolin ＋ ceftazidime to ceftazidime	No	Cured

A distinguishing feature of our case was the lack of subjective symptoms besides cloudy PD effluent, unlike in previous reports. The reason for this remains unclear; however, it suggests that PD-related peritonitis caused by *M. osloensis* may manifest as a wide range of symptoms, indicating that classic symptoms, such as abdominal pain and fever, may not always be present. Additionally, the infection route of *M. osloensis* in PD-related peritonitis has not been thoroughly investigated. In our case, there were no signs of infection at the PD catheter exit site or in the tunnel, and abdominal computed tomography did not indicate an endogenous cause. Considering the presence of *M. osloensis* in the mucosa, touch contamination may be a likely route of infection.

Regarding risk factors for PD-related peritonitis caused by *M. osloensis* in our patient, there were no immunocompromising conditions other than renal failure. Considering that PD-associated peritonitis caused by *M. osloensis* has been reported in the past, it may be better to consider that the immunosuppressive state due to renal failure is also a risk factor for *M. osloensis* infection. Furthermore, given the challenges in identifying *M. osloensis* via traditional culture methods, undiagnosed cases of PD-related peritonitis caused by *M. osloensis* may have existed. As advanced identification techniques, such as MALDI-TOF MS, become more widespread, *M. osloensis* may emerge as a more frequently recognized pathogen in PD-related peritonitis.

## Conclusions

In conclusion, we report a case of PD-related peritonitis caused by *M. osloensis*. Unlike in previous cases, in our case, there was a delay between the onset and the initiation of antibiotic treatment. However, the patient achieved resolution without the need for catheter removal. With advancements in identification techniques for culture testing, more cases of PD-related peritonitis caused by *M. osloensis* are expected to be reported, and continued case accumulation will be beneficial.
